# Agarose capsules as new tools for protecting denuded mouse oocytes/embryos during handling and freezing-thawing and supporting embryonic development *in vivo*

**DOI:** 10.1038/s41598-017-18365-z

**Published:** 2017-12-20

**Authors:** Hiroaki Nagatomo, Tatsuma Yao, Yasuyuki Araki, Eiji Mizutani, Teruhiko Wakayama

**Affiliations:** 1 COC Promotion Center, University of Yamanashi, 4-4-37 Takeda, Kofu, Yamanashi, Japan; 2 Fuso Pharmaceutical Industries, Ltd., Research and Development Center, Joto-ku, Osaka, Japan; 3 The Institute for Assisted Reproductive Medical Technology, Fujimi, Maebashi, Gunma, Japan; 4 Faculty of Life and Environmental Sciences, University of Yamanashi, 4-4-37 Takeda, Kofu, Yamanashi, Japan; 5 Division of Stem Cell Therapy, Center for Stem Cell Biology and Regenerative Medicine, Institute of Medical Science, University of Tokyo, Minato-ku, Tokyo, Japan; 6 Advanced Biotechnology Center, University of Yamanashi, 4-4-37 Takeda, Kofu, Yamanashi, Japan; 7 Center for Life Science Research, University of Yamanashi, Chuo, Japan

## Abstract

Oocytes without a zona pellucida (ZP) often occur as a result of congenital or operational effects, but they are difficult to handle, and embryonic survival is low. Such zona-free (ZF) oocytes are therefore not used in clinics or laboratories. Furthermore, in the laboratory, removal of the ZP is often necessary for genetic manipulation by viral infection or twin production by blastomere separation, but adverse effects on development have been reported. It would therefore be extremely valuable if the embryo could be covered with a structure similar to that of the ZP. In this study, we sought to determine whether an agarose capsule could serve as a substitute for the ZP. Our results indicate that embryos derived from these agarose capsules were able to develop normally, and could be transplanted to obtain viable offspring, without having to remove the agarose capsule. Furthermore, before compaction, the agarose capsule embryos exhibited good freezing tolerance, and survival rate was extremely high compared to ZF embryos. Thus, agarose capsules represent a valuable tool for utilizing oocytes and embryos that lack a ZP, both in a clinical and livestock setting.

## Introduction

Various developmental engineering methods such as *in vitro* fertilization, intracytoplasmic sperm injection (ICSI), and cryopreservation are applied in the fields of infertility treatment, animal reproduction, and rescue of endangered species. During such processes, oocytes that lack a ZP are often encountered. These denuded oocytes are typically discarded because it is difficult to prevent polyspermy at the time of fertilization and they are difficult to handle without damaging them. Thus, the developmental success of such embryos is reduced^1^. Bronson *et al*. showed that it is possible to produce offspring from mouse oocytes that lack a ZP, but the success rate was low^2–4^. The functions of the ZP during *in vitro* culture include providing physical protection to the oocytes and embryos through to the blastocyst stage, serving as a physical barrier to viral infection^5^, and promoting tight junction formation between blastomeres during compaction^6^. Furthermore, naked one-, two-, and four-cell-stage mouse embryos do not develop when transferred to the oviduct^3,7^.

The ZP is also particularly important during freezing of embryos. In a previous report, no zygotes or two-cell-stage embryos that lacked a ZP survived storage in liquid nitrogen^8^. It is, however, possible for embryos to develop normally without a ZP, and the live birth of twins from autologous ZF oocytes was reported in 2016^9^. On the contrary, for assisted reproduction technology or reproductive biotechnology, the ZP is essential for handling oocytes or embryos with a micromanipulator. In the absence of a ZP, researchers must hold the oocyte cytoplasm directly with the pipette, which requires caution and skill.

In an attempt to cover denuded embryos, the embryos have been embedded in agar by sucking the mixture into a glass pipette^10^. However, this produces tubular-shaped agar, which cannot be used with a micromanipulator. The resulting embryos are also difficult to handle because the solidification temperature of the agar is close to the limit required for embryo survival. Moreover, it is necessary to remove the capsule before embryo transfer, otherwise the embryos cannot hatch^11^. A similar method has been applied in bovine embryos, where the embryos were covered with a mixture of a sodium alginate solution gelled in a calcium chloride solution, termed a microcapsule. This alginate encapsulation of bovine embryos does not affect *in vitro* development up to the blastocyst stage, but does significantly hinder the hatching process^12^. Moreover, the microcapsule does not have the same shape as the ZP, and is therefore not suitable for manipulation with a traditional micromanipulator.

Recently, Araki *et al*. produced an agarose capsule that can be used to store sperm individually^13^. The shape of the agarose capsule is extremely similar to that of the ZP, and it is possible to create a capsule with a ZP-like size. In the present study, we sought to determine whether these agarose capsules could be used as a substitute for the ZP. After placing oocytes into the agarose capsules, we examined embryogenesis after ICSI and assessed the freeze-resistant properties of the resulting embryos.

## Results

### Operability of agarose capsules

**Figure 1:**
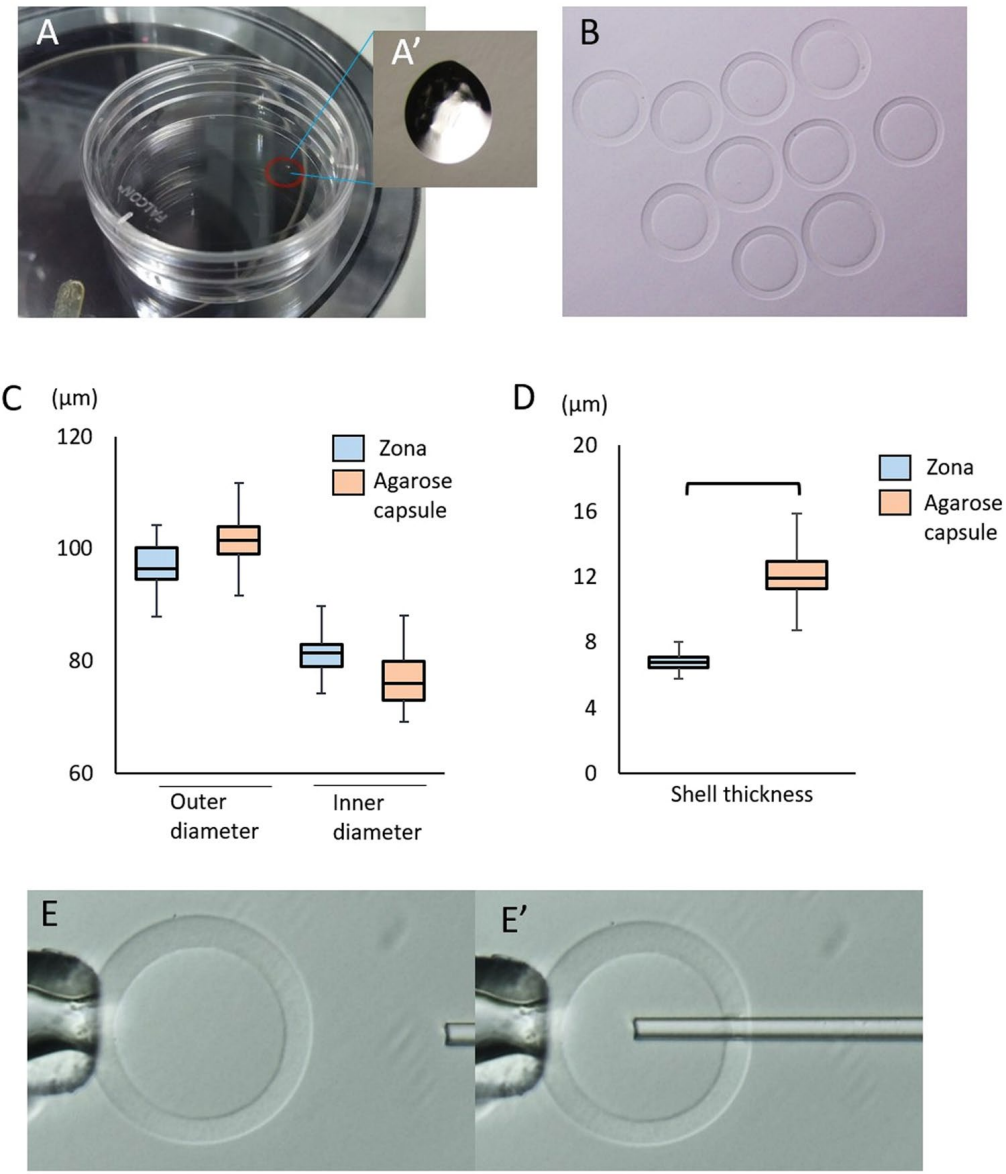
Morphological dimension of agarose capsules. (**A**) Agarose capsules were stored semi-dried in microdroplets consisting of 0.95 M sucrose and 0.05% methyl cellulose. (A′) Enlarged photograph of microdroplets. (**B**) Medium is added and capsules are washed several times. (**C**) Size of agarose capsules. (**D**) Shell thickness of agarose capsules. (**E**, E′) Agarose capsules can be drilled with a glass pipette attached to a piezo driving unit.

**Figure 2:**
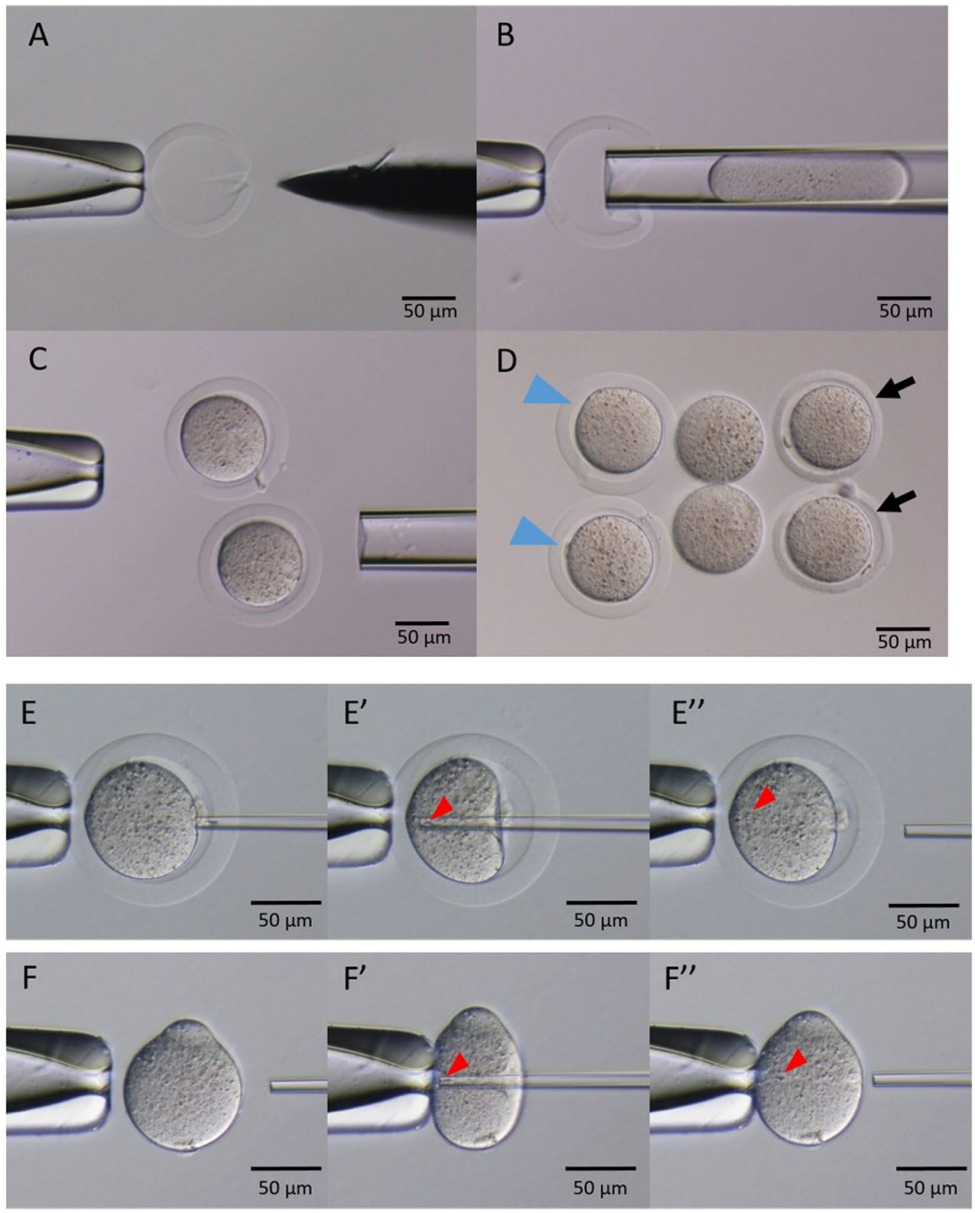
Procedures for injecting denuded oocytes into agarose capsules and intracytoplasmic sperm injection. (**A**) Slit made in agarose capsule with a microblade. (**B**,**C**) Denuded oocytes injected into agarose capsules. (**D**) Morphological comparison of oocytes within agarose capsules, denuded oocytes, and intact oocytes. (**E**, E′, E″) Intracytoplasmic sperm injection of agarose capsule oocytes. (**F**, F′, F″) Intracytoplasmic sperm injection of denuded oocytes. Blue arrowhead, oocyte placed into agarose capsule. Arrow, intact oocyte. Red arrowhead, sperm head.

The agarose capsules were stored in sucrose and methyl cellulose solution; therefore, they were washed several times with CZB medium and incubated in fresh medium for 1 h before use (Fig. 1A,B). First, we studied the form of the agarose capsules. On average, the thickness of agarose capsules was greater than that of the ZP, and the variation was greater (12.1 ± 1.6 vs. 6.8 ± 0.5 µm) (Fig. 1C,D). It was possible to drill into the agarose capsules with a glass pipette attached to a piezo-electric pipette driving unit (Fig. 1E,E′). The agarose capsules had the same operability with a micromanipulator as a normal ZP, and it was possible to cut the capsule with a blade or drill a hole with a piezo drive (Fig. 2A). However, as the thickness of each agarose capsule was slightly different, there was some variation in operability.

### Developmental ability of embryos within agarose capsules

ZF oocytes were carefully sucked into a 40-μm-diameter needle and transferred into the agarose capsules (Fig. 2B–D). The success rate of placing oocytes into the capsules in the presence of CB was 100%, whereas for oocytes in agarose capsules without CB it was 26%, and the remaining 74% suffered a collapsed cytoplasm (Table 1).

**Table 1:** Success rate of placing oocytes into agarose capsules with or without CB.

	No. oocytes	No. placed in agarose capsules	No. survived after incubation (%)
CB+	35	35	35 (100)*
CB−	35	11	9 (26)

CB: cytochalasin B. *P < 0.05.

**Figure 3:**
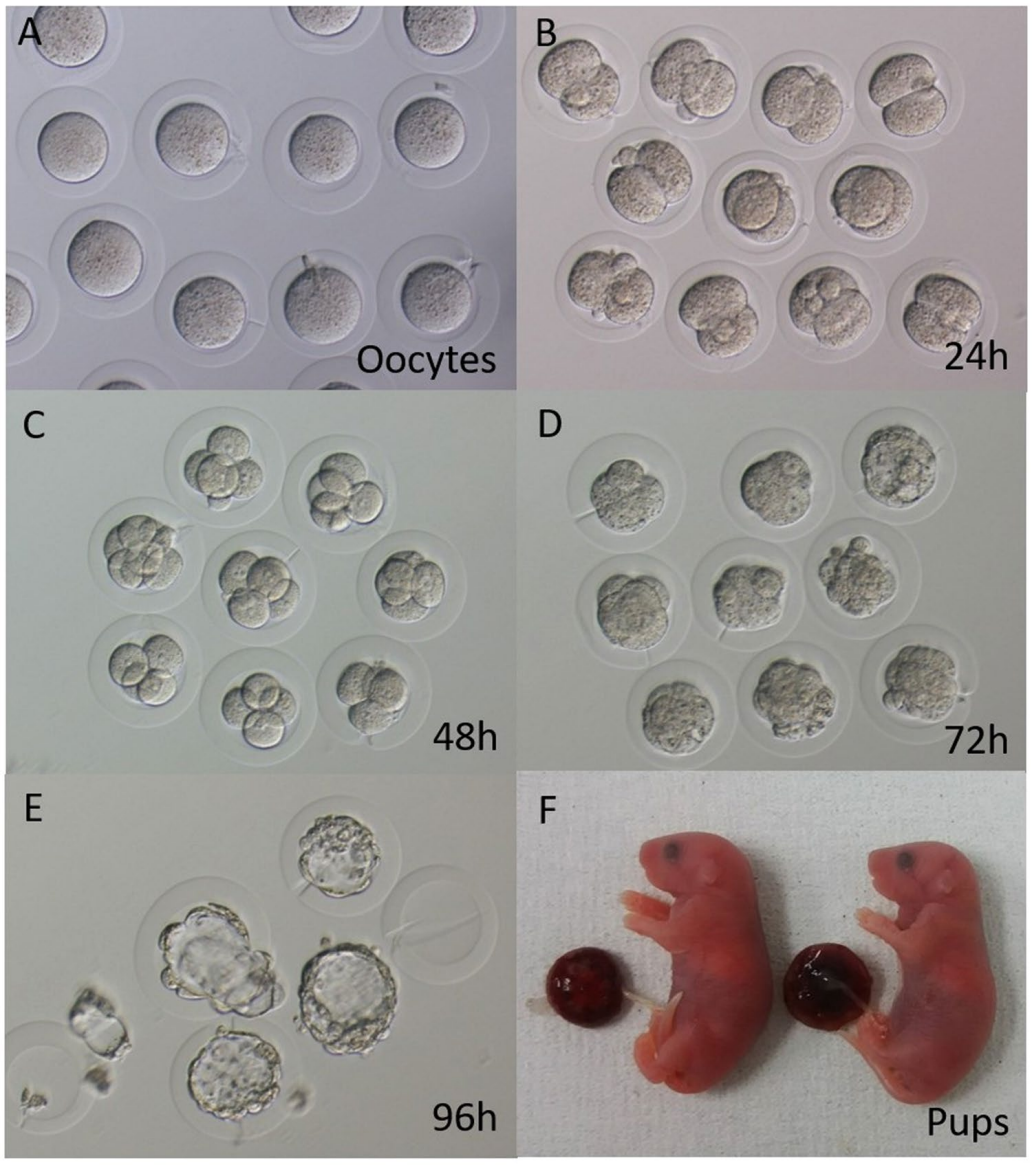
Embryo development in agarose capsules. Oocytes within agarose capsules can be fertilised and develop into blastocysts with a similar success rate as intact controls. (**A**) Oocytes cultured for 30 minutes after placing into agarose capsules. (**B**–**E**) Agarose capsule embryos cultured for 24 hours (**B**), 48 hours (**C**), 72 hours (**D**), 96 hours (**E**) after ICSI. (**F**) Pups obtained after culturing agarose capsule embryos to the blastocyst stage followed by uterine transplantation.

There was no difference in embryo survival rate after ICSI using ZF oocytes versus oocytes within agarose capsules (Fig. 2E,F, and Table 2). However, more embryos within agarose capsules than ZF embryos developed to the blastocyst stage *in vitro* (72% vs. 52%) (Fig. 3). In addition, after 5 days of culture, the hatch rate for agarose capsule embryos was 72%, versus 81% for intact embryos.

**Table 2:** Comparison of developmental rate of agarose capsule and zona-free embryos.

	No. oocytes	No. survived after ICSI	PN	2-cell (24 h)	4–8-cell (48 h)	Morula/blastocyst (72 h)	Blastocyst (96 h) (%)	Hatched blastocyst (120 h) (%)
Agarose capsule	32	26	25	25	24	22	18 (72)	18 (72)
Zona−	33	24	23	23	21	14	12 (52)*	—
Zona+	30	27	26	25	25	24	23 (88)	21 (81)

ICSI: intracytoplasmic sperm injection; PN: pronuclei, *P < 0.05.

To investigate whether agarose capsule embryos hatched in the uterus without assistance, we transferred two-cell-stage embryos into the oviduct of pseudo-pregnant ICR females at 0.5 dpc or transferred morulae/blastocysts into the uterus at 2.5 dpc (Table 3). We found that the mice gave birth to live pups, which displayed a normal developmental ability (Fig. 3). For ZF embryos at the two-cell stage, no pups were obtained following embryo transfer into the oviduct.

**Table 3:** Effect of agarose capsules on full-term development of embryos.

	No. oocytes	No. survived after ICSI	No. PN	No. 2-cell (%/PN)	No. ET at 2-cell	No. 4–8 cell (%/PN)	No. morulae/blastocyst (%/PN)	No. ET at morulae/blastocyst	No. live offspring (%/ET)
Agarose	40	35	35	34 (97)	34	—	—	—	19 (56)
Zona−	40	30	30	30 (100)	30	—	—	—	0*
Zona+	34	29	29	28 (97)	28	—	—	—	11 (40)
Agarose	36	32	31	30 (97)	—	30 (97)	28 (90)	28	13 (46)
Zona−	37	31	30	29 (97)	—	27 (90)	20 (67)*	20	7 (35)
Zona+	36	32	32	32 (100)	—	32 (100)	31 (97)	31	14 (45)

ICSI: intracytoplasmic sperm injection; PN: pronuclei; ET: embryo transfer, *P < 0.05.

### Freezing tolerance of agarose capsule embryos

**Figure 4:**
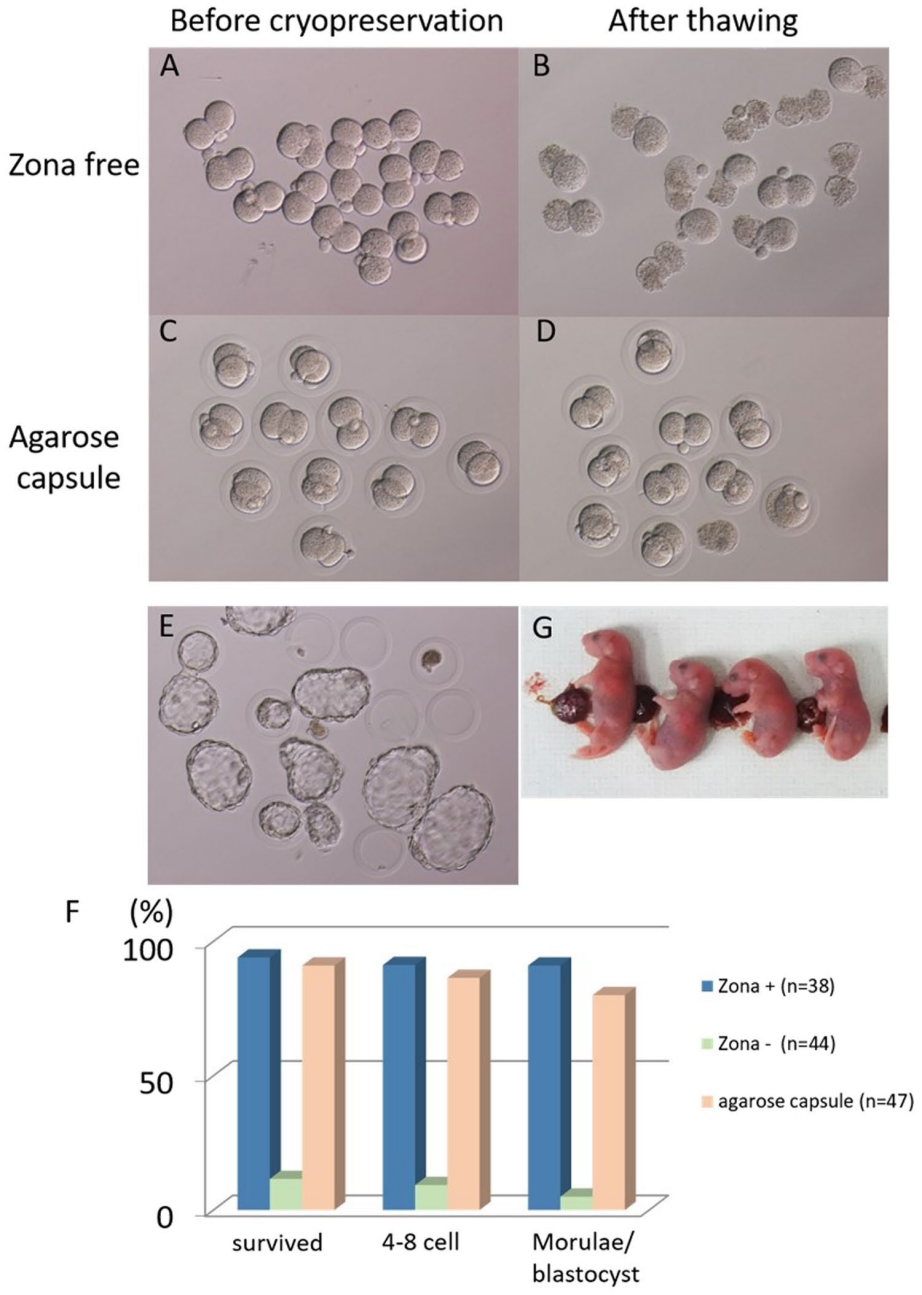
Embryos in agarose capsules exhibit improved freezing resistance. (**A**) ZF two-cell-stage embryos before cryopreservation. (**B**) ZF two-cell-stage embryos after thawing. (**C**) Two-cell-stage embryos within agarose capsules before cryopreservation. (**D**) Two-cell-stage embryos within agarose capsules after thawing. (**E**) Thawed agarose capsule embryos cultured to blastocyst stage. (**F**) Comparison of survival and developmental rates after thawing for agarose capsule, ZF, and intact embryos. (**G**) Offspring obtained after transfer of thawed agarose capsule embryos into the oviduct.

Finally, to investigate whether agarose capsule embryos can develop normally after freezing and thawing, we examined the effect of freezing at the two-cell stage (Fig. 4A–D). When frozen and thawed at the two-cell stage, ZF embryos had survival rate of 5–18% (Fig. 4F and Table 4). Conversely, almost all agarose capsule embryos survived this process. In a few cases, the embryo leaked out of the capsule (approximately 5% of the total), and did not survive. In addition, when the surviving embryos were cultured, they developed to the blastocyst stage with the same success rate as controls, and embryos within agarose capsules had an equivalent hatchability to control embryos (Fig. 4E,F). To investigate their full-term development ability, two-cell-stage embryos from each group were transferred into the oviduct of pseudo-pregnant ICR female mice (Table 4). Whereas no offspring were obtained from ZF two-cell-stage embryos, live, healthy offspring were obtained from two-cell-stage embryos within agarose capsules (44%) (Fig. 4G).

**Table 4:** Comparison of survival rates of two-cell-stage embryos within agarose capsules and zona-free embryos after freeze-thawing.

	No. cryopreserved at 2-cell-stage	No. survived after thawing (%)	No. transferred at 2-cell-stage	No. live offspring (%/ET)
Agarose	42	39 (93)	39	17 (44)
Zona−	151	27 (18)*	27	0*
Zona+	40	36 (90)	36	19 (53)

*P < 0.05.

## Discussion

This study represents the first demonstration that agarose capsules can be used as a substitute for the ZP in ZF oocytes. The capsules can be manipulated with a micromanipulator in a manner similar to that of intact oocytes, and it is possible to obtain offspring after direct implantation without removing the agarose capsule. Furthermore, compared with the extreme difficulty of freezing ZF embryos at the two-cell stage, embryos placed in agarose capsules can be easily frozen.

Using a micromanipulator becomes more difficult as cell size increases, but even oocytes, the largest of cells, can be placed into agarose capsules. Needle size is very important during this step: if the diameter of the needle is too small, ZF oocytes will be damaged when taken up into it, whereas if it is too large it becomes difficult to place into an agarose capsule. As a result, a needle diameter of 40–50 μm was found to be most effective. Addition of CB, which inhibits network formation by actin filaments, improved the operability of the oocyte, and almost all oocytes could be placed into agarose capsules after CB treatment. Although encapsulation of oocytes was possible without adding CB, the success rate was low. Importantly, treatment with CB did not affect the survival rate of embryos compared to controls, indicating that the treatment is not toxic. The size of the slit in the agarose capsule is also important. If the slit is small, the needle cannot enter the capsule, making it difficult to position the oocyte inside. Conversely, if the slit is large, the embryo can escape from the capsule during subsequent operations. Therefore, the size of the slit should be close to the radius of the oocyte. If there was a way to make a larger slit in the agarose capsule then re-seal it after placement of the embryo, these problems could be resolved without the use of CB, but such a method has not yet been developed.

In freeze-thaw experiments, two-cell-stage ZF embryos had a low survival rate, whereas almost all blastocyst-stage embryos survived freezing and thawing, with a survival rate similar to that of control embryos. This result is consistent with those from previous studies^8^. The conception rate after freezing and thawing is low for bovine ZF embryos, indicating that the ZP is indispensable for cryopreservation^14^. In addition, the ZP protects embryonic cells from physical damage associated with the growth of ice crystals during the freezing process^14^. We found that two-cell-stage embryos within agarose capsules exhibited a high survival rate. However, as the vitrification solution is toxic and has adverse effects on embryonic development, it is necessary to remove it promptly after thawing. The mesh-like structure of agarose^15^ may make it suitable for freezing because the capsule physically protect the embryo from ice crystals, while allowing rapid penetration of the cryoprotectant solution and washing as necessary.

In the current study, we placed ZF embryos into agarose capsules and obtained offspring after transplanting two-cell-stage embryos, with or without vitrification, into the oviduct. In previous reports, ZF embryos before compaction did not produce offspring when transplanted into the oviduct^3,7^. The ZP is necessary for implantation of cleavage-stage embryos in the oviduct during normal pregnancy^4^. There has been no research into the intensity of the microvillar movement that moves embryos within the oviduct. When early-stage ZF embryos are placed into the oviduct, the blastomeres are separated by the action of microvilli, and the embryos cannot develop. However, when an agarose capsule embryo is placed into the oviduct, the embryo remains within the capsule, even when the slit in the capsule is large. Therefore, the movement of microvilli in the oviduct is not of sufficient intensity to open the slit in the agarose capsule. By adjusting the thickness of the agarose capsule, it may be possible to examine the effect of microvillar movement on embryonic development.

In addition to the importance of the size of the slit in the agarose capsule, our experiments showed that the slit has to be sufficiently supple to allow the embryo to hatch when it enters the uterus and forms an expanded blastocyst. Previous reports showed that while other artificial ZPs were able to cover the embryo, they had to be removed in order to produce offspring after transplantation. Our method requires time to carefully place the oocyte into the agarose capsule, and CB treatment is necessary to increase the success rate of this process. However, the oocyte is not damaged, and transplantation can be done at the two-cell stage, making the strategy more advantageous than conventional methods.

The generation of transgenic mice using lentivirus is highly efficient, and placenta-specific gene manipulation is also possible using this method, making it useful for gene therapy^16^. Removal of the ZP is necessary prior to virus infection, but this is thought to have adverse effects on embryogenesis. It has also been reported that twin production in mice via blastomere separation is very difficult due to an inability to maintain a normal conformation at the four-cell stage after removing the ZP^17^. Using agarose capsules may thus improve the efficiency of transgenic mouse production and twin production.

In the present study, progeny were obtained by transplanting agarose capsule embryos at the two-cell stage, and freezing resistance was dramatically improved compared to ZF embryos. Agarose capsules can thus be used as a substitute for the ZP. Despite the difficulty of placing the embryo into the agarose capsule, the technique may represent an important resource to avoid wasting oocytes and may also be useful for efficient production of identical twins in livestock animals.

## Methods

### Animals

B6D2F1 (C57BL/6 N × DBA/2) female and male mice, 8–10-weeks-old, were used to produce embryos. Female ICR mice that had been mated with vasectomised males of the same strain were used as surrogate pseudo-pregnant recipients for embryo transfer. B6D2F1 and ICR mice were purchased from SLC Co. Ltd. (Shizuoka, Japan). On the day of the experiment or after finishing all experiments, mice were euthanised via CO_2_ inhalation or cervical dislocation. All animal experiments conformed with the Guide for the Care and Use of Laboratory Animals, and were approved by the Institutional Committee of Laboratory Animal Experimentation of the University of Yamanashi.

### Agarose capsules

Agarose capsules were produced according to the method described in a previous study^13^. Briefly, alginate beads were prepared using 0.5% calcium carbonate and 4% alginate acid solutions. Alginate beads were placed into a 2% (wt/vol) agarose solution. The agarose solution containing the alginate beads was mixed with mineral oil, and agarose gel spheres were formed by cooling the solution on ice. Finally, the alginate beads in the agarose gel were dissolved in 50 mM sodium citrate solution. The resulting agarose capsules were placed into microdroplets composed of 0.95 M sucrose and 0.05% methylcellulose solution on the bottom of the dish and embedded semi-dried, before being sterilized with gamma rays (approximately 12 capsules per dish) (Fig. 1A,B). These agarose capsules are being prepared for commercial sale, but at present are available to researchers on request. Before use, 50 µl HEPEZ-CZB medium^18^ was added to the semi-dried agarose droplets. The agarose capsules were washed several times then incubated in HEPEZ-CZB medium covered with mineral oil at room temperature for at least 1 hour. This allowed exchange of the solution within the capsules with the HEPEZ-CZB medium. To measure their diameter and thickness, we captured images at 400 times magnification using an inverted microscope and Olympus Cell Sens imaging software. Captured images were further magnified 4 times using the digital zoom function within the software, and measured using a dimension measuring tool. Measurements were obtained from 45 ZP and 48 agarose capsules. To determine whether the agarose capsules could be manipulated with a micromanipulator, we attempted to pierce them using a piezo drive (Fig. 1C–E′).

### Metaphase II (MII) oocyte collection

Female mice were induced to undergo superovulation via treatment with 5 IU pregnant mare serum gonadotropin (ASKA Pharmaceutical Co., Ltd., Tokyo, Japan) followed 48 hours later by 5 IU human chorionic gonadotropin (hCG) (ASKA Pharmaceutical Co., Ltd.). Sixteen hours after hCG treatment, cumulus–oocyte complexes (COCs) were collected from the oviducts as quickly as possible and placed into HEPES CZB medium containing 0.1% bovine testicular hyaluronidase (Sigma-Aldrich, St Louis, MO, USA). After several minutes, oocytes were washed and placed into a droplet of CZB medium for culture.

### Oocyte place into agarose capsules

The ZP was removed from oocytes in acidic Tyrode’s solution (pH 2.5). Briefly, oocytes were placed into 50 μl drops of acidic Tyrode’s solution until the ZP dissolved. The oocytes were then washed three times in CZB medium and incubated for one hour. A slit was made in an agarose capsule using a micromanipulator equipped with a microsurgical blade (Feather, Osaka, Japan) under an inverted microscope (Olympus, Tokyo, Japan) (Fig. 2A). Slit size was set to approximately match the radius of the capsule. ZF oocytes and slit agarose capsules were placed into a droplet of HEPES-CZB containing 5 mg/ml cytochalasin B (CB) on a microscope stage and allowed to stand at room temperature for 10 minutes. ZF oocytes were carefully taken up into a 40-μm-diameter needle and transferred into the capsules. After washing with CZB medium, the reconstructed oocytes were cultured until ICSI.

### Intracytoplasmic sperm injection (ICSI)

ICSI was performed as previously described^19^. The sperm suspension (1–2 μl) was mixed with a droplet of PVP solution. The sperm head was separated from the tail by the application of several piezo pulses, and the head was injected into the oocyte. After ICSI, oocytes were left on the microscope stage for 10 minutes, and those that survived were incubated in CZB medium at 37 °C in an atmosphere of 5% CO_2_ in air. Pronucleus formation was checked 6 hours after ICSI.

### Embryo transfer

To produce offspring, we transferred two-cell-stage embryos or morulae/blastocysts into pseudo-pregnant ICR females that had been mated with a vasectomised male the night before. Transfers were done into the oviduct at 0.5 days post-coitum (dpc) or into the uterus at 2.5 dpc. Offspring were delivered at 18.5 dpc, one day before full term delivery, via caesarean section.

### Embryo cryopreservation and thawing

Embryo cryopreservation and thawing were performed according to a previous study^20,21^. Briefly, vitrification solutions, namely EFS20 and EFS40, which consisted of 20 and 40% (v/v) EG, respectively, in PB1 solution containing 30% (w/v) Ficoll (average molecular weight, 70,000) and 0.5 M sucrose, were prepared. A total of 20–40 embryos were suspended in EFS20 solution for 2 minutes at room temperature, then transferred directly into approximately 50 µl EFS40 solution in a cryotube, as previously described. After exposing the embryos to EFS40 solution at room temperature for 30 seconds, the lid was loosely tightened, and the tube was immersed in liquid nitrogen.

For thawing, 0.25 and 0.75 M sucrose-PB1 solutions were prepared. The cryotube was removed from liquid nitrogen, then opened and left to stand at room temperature for 30 seconds. Eight-hundred microlitres of 0.75 M sucrose-PB1 was added to the tube and slowly pipetted up and down 10 times. The contents of the tube were transferred to a dish and incubated at room temperature for 3 minutes. Embryos were transferred to 0.25 M sucrose-PB1 and allowed to stand for 3 minutes, then washed and placed into a droplet of CZB medium for culture.

### Statistical analysis

Capsule diameter and thickness were evaluated using independent t tests, and P < 0.05 was considered statistically significant. Developmental and offspring rates were evaluated using a chi-square test, and P < 0.05 was considered statistically significant.
